# The effect of quality culture on service quality of public and private Universities: A comparative analysis

**DOI:** 10.1371/journal.pone.0283679

**Published:** 2023-04-13

**Authors:** Shahzaf Iqbal, Tahir Ashfaq, Che Azlan Bin Taib, Mohd Rizal Razalli

**Affiliations:** 1 School of Technology Management and Logistics, Universiti Utara Malaysia, Sintok, Kedah, Malaysia; 2 School of Business Management, Universiti Utara Malaysia, Sintok, Kedah, Malaysia; University of Technology Malaysia, MALAYSIA

## Abstract

This study aims to differentiate between perceived quality culture (QC) and service quality (SQ) in public and private universities; and to examine the effect of QC on SQ in both types of universities, individually and collectively. This is a quantitative study in which data are collected from administrative and quality managers of randomly selected universities in Pakistan through face-to-face and online surveys. Of the 150 questionnaires distributed, a total of 111 questionnaires are received, of which 105 are valid, giving a response rate of 70%. The collected data are then analyzed by descriptive and causal research methods using SPSS-25 and PLS-SEM. The findings reveal significant differences in perceived QC and SQ between public and private universities; however, public universities scored higher on both variables than private universities. Furthermore, the results show the significant effect of QC on SQ individually and collectively in public and private universities; however, this relationship is stronger for private universities than for public universities. The findings of the study may help administrative and quality managers to improve SQ by cultivating QC in their respective universities, thereby improving organizational performance. This study extends theoretical knowledge by introducing QC as a predictor variable and then measuring SQ from a dual perspective (internal and external customers) in a university setting, which is less explored in the existing literature.

## 1. Introduction

In the first two decades of the 21st century, universities have multiplied worldwide, and this is also evident in Pakistan, resulting in a highly competitive environment. In such an environment, some authors emphasize that institutions must employ various marketing strategies to attract students and gain a competitive advantage [1]. In contrast, others emphasize that universities need to restructure their quality systems and adhere to national and international standards [2]. While profitability and survival are often considered the primary goals of any institution, they should not come at the expense of academic quality. Given such concerns, leaders, and administrators of higher education institutions (hereinafter HEIs) must adopt proactive coping strategies without compromising quality.

Creating and promoting a quality culture (hereinafter QC) in HEIs is one such strategy. QC is a continuous improvement process in which the entire organizational community is responsible for maintaining a conducive work environment leading to organizational excellence [3]. In an educational context, researchers have defined QC as “the overall attitude of an institution, which focuses on the concept of quality and applies it to all aspects of its activities” [4]. Some researchers have primarily investigated QC as a predictor variable and found it to be positively associated with organizational performance [4–7]. Likewise, a few studies have attempted to examine culture related to SQ, but mainly in hospitality [8], public sector services [9], the power sector [10], information service functions [11], and Romania’s ICT sector [12]. However, these studies attempted to investigate the relationship between OC (rather than QC) and SQ outside of educational settings. Thus, there are theoretical and empirical gaps in the current literature.

Another important strategy for service-oriented organizations is to provide the best quality of services and HEIs are no exception. Service quality (hereinafter SQ) is considered an integral part of the performance of any university worldwide [13]. In Pakistan, the quality of HE is often criticized by academics across the country. Particularly, researchers have identified three key areas of quality deficits, such as teaching and research [14, 15], and other related services, like academic programs [16–18]. However, the role of services is relatively more comprehensive and all-encompassing. For example, the “International Organization for Standardization”, commonly referred to as "ISO", defines educational service as the “process that supports acquisition and development of learners’ competence through teaching, learning or research" [19]. According to this definition, educational services include all processes of developing students’ skills, including teaching, learning, and research, so all processes should be given equal consideration as part of educational services in the educational environment. Likewise, SQ tends to increase student satisfaction [20–24], and student loyalty [22, 23, 25], which in turn improves university performance [24, 26–28].

The conceptualization of SQ is difficult in higher education [29–32], probably due to the presence of multiple stakeholders [19, 33–35], their different needs and preferences [36], and due to some unique characteristics [37, 38], which makes it difficult to define SQ uniformly. In order to better serve different types of stakeholders, the researchers divided customers and related services into two categories, internal customer service, and external customer service [39, 40]. The former category includes services provided to internal customers or employees of one unit to employees of another unit within the same organization [41–45], while the latter category refers to services provided by organizations to external customers [43, 44].

Researchers have equally emphasized two types of services and suggested combining the two when trying to measure SQ [46]. However, so far, most of the previous studies have examined SQ from the perspective of external customers [20, 22–24, 47–58], and only a few studies have examined both aspects of SQ at the same time [59–63]. Thus, there is an empirical gap in the existing literature. Given the subjectivity and different perspectives of SQ, and the involvement of multiple stakeholders; following two research questions (RQs) have been proposed:

RQ1: Are there any differences in perceived quality culture and service quality between public and private universities?
RQ2: What is the effect of quality culture on service quality between public and private universities?

Therefore, the purpose of this study is to differentiate the perceived QC and SQ of public and private universities and examine the individual and collective effects of QC on the SQ of both types of universities. The novelty of this study is threefold: First, this study investigates perceived QC and SQ to understand their current state and compares them in the context of universities (public and private), which is lacking in the existing literature. Second, the study operationalizes SQ from a dual perspective, which includes internal service quality (hereinafter ISQ), which includes services provided to university staff (academic and non-academic staff); and external service quality (hereinafter ESQ), which includes services to students and the community. Lastly, there is little evidence that researchers have examined QC as a predictor of SQ in previous literature; therefore, this study fills this gap by examining QC in relation to SQ based on two perspectives (including administrative and quality managers) in the university environment.

In terms of paper structure, following the introduction, a literature review is presented with the development of the hypotheses. Then, research methods are discussed, including sampling, data collection, and measurement. Subsequently, results, discussions, and conclusions are presented, along with recommendations for the future.

## 2. Literature review

### 2.1 Quality culture

Over the last two decades, the notion of QC as part of organizational culture (OC) [64] has received much attention from researchers. However, despite wider acceptance, its meaning remains ambiguous in earlier literature [2]. QC in a broader context is “a system of shared values, beliefs and norms that focuses on delighting customers and continuously improving the quality of products and services” [65]. This definition has focused on two aspects, first to delight customers and second to constantly improve the quality of products and services. In the context of HE, the first and foremost definition of a QC, which has gained wide acceptance as part of the OC, is as follows:

*“Quality culture refers to an organizational culture that intends to enhance quality permanently and is characterized by two distinct elements*: *on the one hand*, *a cultural/psychological element of shared values*, *beliefs*, *expectations and commitment towards quality and*, *on the other hand*, *a structural/managerial element with defined processes that enhance quality and aim at coordinating individual efforts*” [66].

The EUA definition of QC consists of two aspects: the first aspect demonstrates the cultural/psychological components (e.g., values, beliefs, expectations, etc.) which are generally difficult to capture, while the second aspect is “structural/managerial” that focuses on different processes and efforts to achieve and improve quality. Despite the EUA definition of QC, the authors continued to explore the concept of QC from different perspectives, such as QC is “a set of shared, accepted, and integrated patterns of quality (often called principles of quality) to be found in the organizational cultures and the management systems of institutions” [67]. Also, in another definition, researchers have very concisely defined QC as “the overall attitude of an institution, which focuses on the concept of quality and applies it to all aspects of its activities” [4]. Although, researchers have argued that the importance of a QC is equally important for all kinds of organizations, be they schools or universities, commercial or non-profit entities, and even government organizations [68]. However, there is little evidence that researchers have conducted comparative studies involving QC in an organizational context with the specific aim of examining the prevailing state of perceived QC in public and private universities worldwide. Therefore, there are theoretical and empirical gaps in the existing literature.

**H1.** There is a difference in perceived quality culture between public and private universities.

### 2.2 Service quality

Services are an integral part of any organization, on the one hand, their role in product manufacturing organizations is secondary or auxiliary, whereas, in service industries, their role is dominant or primary due to the nature of their core business. Service can be defined as the “detailed description of what is to be done for the customer (what needs and wishes are to be satisfied) and how this is to be achieved” [69]. HEIs are also part of the service industry, whose core mission is to provide quality services (academic and non-academic) to different stakeholders, and any compromise on SQ may lead to dissatisfaction among stakeholders.

Despite the critical role of SQ in HEIs, there is still no common definition or model to measure SQ. Consequently, some researchers called SQ an "elusive" concept [29, 30]; while others considered it "unsolved" [31]; and "far from conclusive" [32]. This may be due to the involvement of multiple stakeholders. Such as, students, parents, academic and non-academic staff, employers, governments, funding/sponsoring bodies, accrediting bodies, auditors, employers, and society [34, 35]. Due to the ambiguity of the variety of stakeholders, researchers defined SQ from different perspectives, including perceptions versus expectations [47]; while others perceived it as performance versus specific standards [70] or only the perceptions about performance [49].

However, all these definitions were from the perspective of external customers [71], until, in the early 1990s, the terms internal customers (employees) and external customers (students) were coined [39]. ISQ is described as the quality of service provided to internal customers or employees of one unit to employees of another unit within the same organization [41–45]. Conversely, ESQ is referred to as the quality of the services that the organization provides to external customers [43, 44]. This is also supported by another study, where a researcher divides these stakeholders into internal and external. External stakeholders or primary customers are outside of the organization, while internal stakeholders are academic and non-academic staff, who are employees of the organization [40].

Unlike external service research, the literature dealing with internal services is relatively limited, and even fewer discuss the dual aspects of services. The researchers have argued that provider and recipient perceptions should be combined when attempting to measure SQ [46]. In another study, researchers have emphasized that meeting the needs of internal customers is a prerequisite for providing high-quality services to end customers [72]. Given the importance of internal services, researchers believe that the quality of external services depends largely on the satisfaction of internal customers. The higher the employee satisfaction, the higher the quality of services provided to external customers [73–75]. In a recent study, researchers have argued that ISQ has the potential to influence service performance [76]. However, despite the importance of this dual perspective of SQ, only a few researchers have examined both aspects of services [59–63], while the emphasis of most of the literature is on the quality of service perceived by the external customer [20, 22–24, 47–58]. Given the involvement of multiple stakeholders, the debates on internal and external customers, and the guidance of previous researchers to measure SQ from a dual perspective, the following hypothesis is proposed:

**H2.** There is a difference in perceived service quality between public and private universities.

### 2.3 Quality culture and service quality

Despite the abundant literature on SQ in the context of HE, most studies examined SQ through an external lens based on the perception of external customers of the university (i.e., students) [21–24, 77, 78]. In contrast, some authors have argued that there are also other stakeholders that are equally important [79], such as parents [80]; faculty members [80, 81]; or internal customers such as employees [43–45]; and employers [82–84]. Therefore, researchers have suggested incorporating other stakeholders in future studies to measure SQ in HE [85].

Also, even though there are numerous studies that have attempted to measure SQ in HE as a predictor variable [21, 23, 58, 77, 86, 87]. However, only a few studies have examined SQ as a criterion (dependent) variable, but mainly in the hospitality industry [8, 88]. Therefore, there are also empirical gaps in the existing literature on SQ as a dependent variable in HE. Additionally, although researchers have investigated OC in relation to SQ; however, these studies were conducted in the public sector [9], the “Information and Communication Technology” (ICT) sector [12], and the power sector [10], but their results have confirmed the significant influence of OC on SQ. Besides, in a relatively new study, researchers found that OC had a strong influence on SQ in Kenyan public and private universities [89]. However, since so far there is no evidence of any research in relation to QC and SQ, especially in a HE context, there are theoretical, empirical, and practical gaps in the previous literature. Given the gaps identified in the literature, three hypotheses have been proposed:

**H3.** Quality culture has a significant effect on the service quality of public universities.
**H4.** Quality culture has a significant effect on the service quality of private universities.
**H5.** Quality culture has a significant effect on the service quality of public and private universities.

### 2.4 Research framework

**Fig 1:**
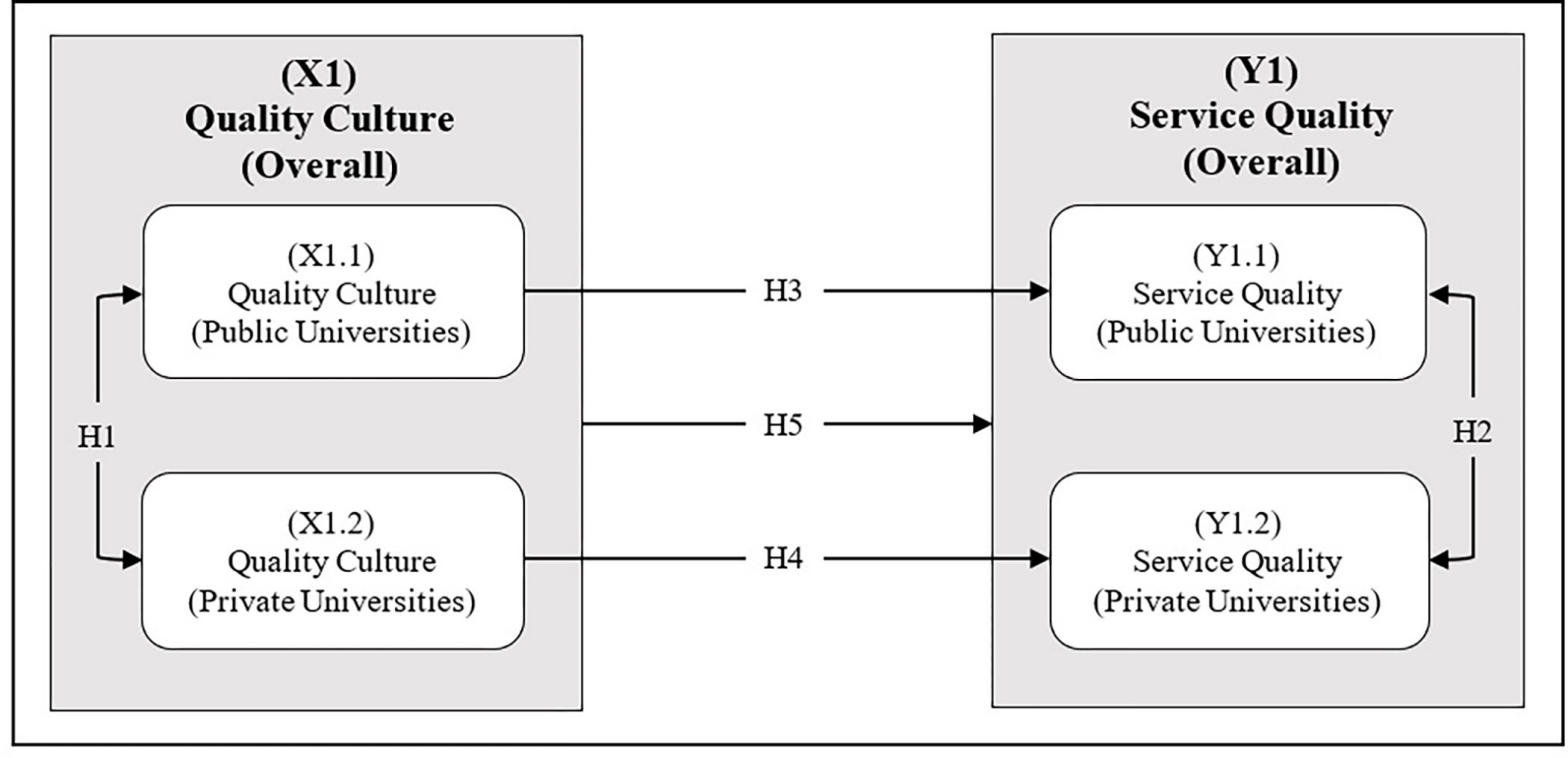
Conceptual framework.

Considering the gaps identified in the extant literature, a conceptual framework based on the Resource-Based View (RBV) theory has been proposed. The RBV theory holds that organizations can gain a sustainable competitive advantage over their rivals by focusing on their strategic resources; however, the resource must be valuable, rare, and cannot be copied and/or replaced [90]. These strategic resources are basically the internal strengths of companies that determine their competitiveness, and which in turn determines their performance [90, 91]. Based on RBV theory, universities can develop and leverage their internal capabilities, such as developing QC, to make university staff (teaching and non-teaching) quality conscious, thus being able to perfect their daily tasks, reduce errors and improve SQ. Keeping this in mind, this study proposes that QC as a valuable resource can bring about major changes by improving the quality of services in both types of universities (public and private) in Pakistan. The framework consists of one independent variable (QC), and one dependent variable (SQ). The conceptual framework is shown below in Fig 1.

## 3 Research methodology

### 3.1 Sample and procedure

This was a quantitative study in which the researchers used SPSS-25 and SEM-PLS 4 software to test the proposed hypotheses. The underlying reasons for choosing PLS-SEM are its suitability for dealing with complex prediction-oriented structural models, handling relatively small sample sizes, and when the data do not follow a normal distribution [92]. The study population was 226 public and private universities in Pakistan, while universities were the unit of analysis, and administrative managers (such as VCs, Deans, Directors, and HODs) and quality managers were the respondents (i.e., the observation unit). In most cases, the "unit of analysis" and the "unit of observation" are similar, but not always [93]. The unit of analysis is defined as the “entity on the basis of which analysis is done” while the unit of observation refers to the “entity at which measurements are done” [94]. Reasons for selecting administrative and quality managers include their organizational knowledge, experience, and their key role in raising quality awareness in universities [95–98]. A sample size of 144 universities was estimated using the tables of Krejcie and Morgan [99]. But to be on the safe side, a total of 150 universities in Pakistan were selected through stratified random sampling techniques, which is often preferred in large-scale surveys because of its multiple advantages, including subpopulation estimation, administrative ease, sample representativeness, efficiency, and improved data quality [100]. Moreover, data were collected through face-to-face and online surveys. For this purpose, the entire population (universities) was divided into seven strata, based on four provinces (including Balochistan, KPK, Punjab and Sindh) and three administrative units (including Azad Kashmir, Gilgit Baltistan and Islamabad) of Pakistan. A total of 150 questionnaires were distributed, of which 111 were returned and 105 were found to be valid, with a response rate of 70.0%.

### 3.2 Measures

The questionnaire used for data collection was divided into two parts, the first part had 09 demographic questions and the second part had 19 questions. QC was measured using four questions, as found in previous literature [6]. SQ was measured using 15 questions for two first-order constructs, comprising seven ISQ questions and eight ESQ questions adapted from various studies [101–105]. This study was conducted with the prior approval of the Postgraduate Studies Unit, Universiti Utara Malaysia (Ref. UUM/COB/P-40). Furthermore, written informed consent was obtained from all participants before administering the questionnaires through face-to-face and online survey methods. Besides, an ethics statement was added to the top of the questionnaire stating that participation in the survey would be purely voluntary, and all participants were assured that the data collected would be kept confidential. Prior to data collection, the questionnaire was validated by consulting six experts, including senior faculty and TQM practitioners from leading universities, and then refined based on their feedback. The questionnaire was of a 5-point Likert type, with 1 = "strongly disagree" and 5 = " strongly agree".

## 4 Data analysis

### 4.1 Demographic characteristics

The demographic characteristics of respondents are presented in Table 1, which indicates that 59 (56.2%) of the respondents were from public universities and 46 (43.8%) from private sector universities. In terms of provinces, most universities 36 (34.3%) were from Punjab, followed by 23 (21.9%) from Sindh, 20 (19%) from Khyber Pakhtunkhwa, 17 (16.2%) from Islamabad, 5 (4.8%) from Balochistan, 3 (2.9%) from Azad Jammu Kashmir, and 1 (1%) from Gilgit Baltistan, respectively. Regarding gender, 81 (77.1%) were male, and 24 (22.9%) were female. Furthermore, most respondents 67 (63.8%) were HODs, while 17 (16.2%) were Deans and 13 (12.4%) were QEC directors, followed by respondents with other designations.

**Table 1:** Demographic characteristics of respondents.

Items	Public		Private		Total	
	Frequency	Percent	Frequency	Percent	Frequency	Percent
**Sector**	59	56.2	46	43.8	105	100
**Province**						
Azad Jammu Kashmir	2	3.4	1	2.2	3	2.9
Balochistan	5	8.5	**-**	**-**	5	4.8
Gilgit Baltistan	1	1.7	**-**	**-**	1	1.0
Islamabad	10	16.9	7	15.2	17	16.2
Khyber Pakhtunkhwa	17	28.8	3	6.5	20	19.0
Punjab	16	27.1	20	43.5	36	34.3
Sindh	8	13.6	15	32.6	23	21.9
**Gender**						
Male	46	78.0	35	76.1	81	77.1
Female	13	22.0	11	23.9	24	22.9
**Designation**						
VC	3	5.1	1	2.2	4	3.8
Dean	9	15.3	8	17.4	17	16.2
Director QEC	4	6.8	9	19.6	13	12.4
Director ORIC	1	1.7	1	2.2	2	1.9
HOD	40	67.8	27	58.7	67	63.8
Other	2	3.4	**-**	**-**	2	1.9

Source: Authors’ own findings.

### 4.2 Data normality

Before performing inferential statistical analysis, the data were first subjected to the Shapiro-Wilk test to check the normality of the data. Since the p-value was found to be less than 0.05, H0 was rejected; implying that the data were not normal.

### 4.3 Common-method bias

The data were also analyzed for CMB, as recommended by previous researchers [106]. A “Full Collinearity Test” was used for CMB detection and the resulting VIF values (Table 2) were found to be below the threshold of 3.3 recommended by [107]. This means that there was no CMB problem, and the results were free of any bias.

**Table 2:** Full-collinearity test results.

Criterion Variable	Predictor Variables	Tolerance	VIF
ISQ	ESQ	0.573	1.745
	QC	0.573	1.745
ESQ	ISQ	0.593	1.686
	QC	0.593	1.686
QC	ISQ	0.315	3.176
	ESQ	0.315	3.176

Abbreviations: QC, quality culture; ISQ, “internal service quality”; ESQ, “external service quality”.

### 4.4 Measurement model assessment

The evaluation of the measurement model was carried out with several purposes, namely, to determine the factor loadings, the reliability (internal consistency), and the validity (convergent and discriminant) [108, 109]. The factor loadings of all items were found to be greater than 0.600, except ISQ3, ISQ7, ISQ8, and ISQ9, which had factor loadings below the cut-off value of 0.500 and were therefore removed [108]. Similarly, internal consistency was tested through composite reliability (CR) as suggested [110], and convergent validity through “Average Variance Extracted” or simply AVE values of the constructs. Overall, the results (Table 3) indicated that the values of Alpha and CR were greater than 0.700 [111] as well as the values of the AVE of all the constructs were greater than 0.500 [108, 109], so they were all considered acceptable. Similarly, discriminant validity, which is “the extent to which a construct is truly distinct from other constructs by empirical standards” [108], was assessed using the “Heterotrait-Monotrait Ratio” (HTMT). The resulting HTMT ratio (Table 4) was found to be less than 0.85 [112], so it was also considered acceptable.

**Table 3:** Factor loading, reliability, and convergent validity.

Constructs	Item	Loading	Alpha	rho_A	CR	AVE
Quality Culture	QC1	0.815	0.862	0.870	0.906	0.707
	QC2	0.858				
	QC3	0.849				
	QC4	0.840				
Service Quality	ISQ1	0.780	0.877	0.886	0.942	0.890
	ISQ2	0.793				
	ISQ4	0.823				
	ISQ5	0.864				
	ISQ6	0.869				
	ESQ1	0.843				
	ESQ2	0.833				
	ESQ3	0.867				
	ESQ4	0.881				
	ESQ5	0.863				
	ESQ6	0.715				

Abbreviations: AVE, average variance extracted; CR, composite reliability.

**Table 4:** Discriminant validity—HTMT ratio.

	QC	SQ
**QC**		
**SQ**	0.759	

Abbreviations: QC, quality culture; SQ, service quality.

### 4.5 Descriptive analysis

The mean QC scores in public and private universities were 3.5847 and 2.9783 respectively. Similarly, the mean SQ scores at public and private universities were 3.5162 and 3.1542 respectively. The results also suggest that the perceived QC and SQ scores (mean) are higher in public universities than in private universities in Pakistan. The sector-wise mean QC and SQ scores are presented in Table 5.

**Table 5:** Sector wise mean scores of QC and SQ.

	Sector	N	Mean	Std. Error	Std. Dev.
**QC**	Public	59	3.5847	.10356	.79548
	Private	46	2.9783	.13022	.88322
	Total	105	3.3190	.08629	.88422
**SQ**	Public	59	3.5162	.07787	.59814
	Private	46	3.1542	.13686	.92826
	Total	105	3.3576	.07590	.77778

Abbreviations: QC, quality culture; SQ, service quality.

### 4.6 Hypotheses testing

A total of five hypotheses (H1-H5) were proposed to answer the two research questions. All hypotheses were tested using SPSS-25 software, except hypothesis (H5), which was also tested using SmartPLS-4.

#### 4.6.1 Mann-Whitney U test

Since the data were found to be abnormal, RQ1 was addressed by testing two hypotheses (H1 and H2) with the Mann-Whitney U test. Both hypotheses aimed to differentiate perceived QC and SQ in Pakistani public and private universities. Hypothesis (H1) aimed to assess the difference in perceived QC in both types of universities in Pakistan. The test revealed substantial differences in the QC perceptions of administrative and quality managers of public (median = 3.50, n = 59) and private universities (median = 2.75, n = 46) in Pakistan, while U = 820, z = 3.482, r = 0.34 and p = 0.000. Therefore, H1 was found to be acceptable. Similarly, hypothesis (H2) aimed to assess the difference in perceived SQ between two types of Pakistani universities. The test revealed considerable differences in SQ perceptions of administrative and quality managers of public (median = 3.53, n = 59) and private universities (median = 3.03, n = 46) in Pakistan, while U = 1001, z = 2.298, r = 0.22 and p = 0.022. Hence, H2 was also found to be acceptable. The results also indicate that the perceived QC and SQ scores (median) are higher in public universities than in private universities in Pakistan.

#### 4.6.2 Regression analysis

Regarding RQ2, three hypotheses were proposed (H3, H4, and H5). Two of them (H3 and H4) were tested using comparative linear regression analysis to examine the effect of QC on SQ in both types of universities in Pakistan individually. Hypothesis (H3) was about testing the effect of QC on SQ in Pakistani public universities. The linear regression test results (shown in Table 6) showed a coefficient of determination (R^2^) of 0.212, which means that the model can only explain 21.2% of the variation of QC to SQ. However, given that the F statistic (15.290), the standardized coefficient (β) (0.460), the t-value (3.910), and the p-value (0.000), which is less than 0.05. H0 was rejected and H3 was accepted. This means that the model is significant. Also, hypothesis (H4) was about testing the effect of QC on SQ among private universities in Pakistan. The linear regression test results (shown in Table 6) showed a coefficient of determination (R^2^) of 0.611, which means that the model can explain 61.1% of the variation of QC to SQ. Given that the F statistic (69.083), the standardized coefficient (β) (0.782), the t-value (8.312), and the p-value (0.000), which is less than 0.05. H0 was rejected and H4 was accepted. This means that the model is significant.

**Table 6:** Comparative linear regression analysis.

**Model Summary**								
**Sector**	**R**		**R Square**	**Adjusted R Square**			**Std. Error of the Estimate**	
Public Sector	.460		.212	.198			.53577	
Private Sector	.782		.611	.602			.58557	
**ANOVA**								
**Sector**		**Sum of Squares**			**Mean Square**	**F**		**Sig.**
Public Sector	Regression	4.389			4.389	15.290		.000^b^
	Residual	16.362			.287			
	Total	20.751						
Private Sector	Regression	23.688			23.688	69.083		.000^b^
	Residual	15.087			.343			
	Total	38.775						

Dependent Variable: Service Quality, Predictor Variable: Quality Culture

### 4.7 Structural Equation Modeling (SEM) analysis

**Fig 2:**
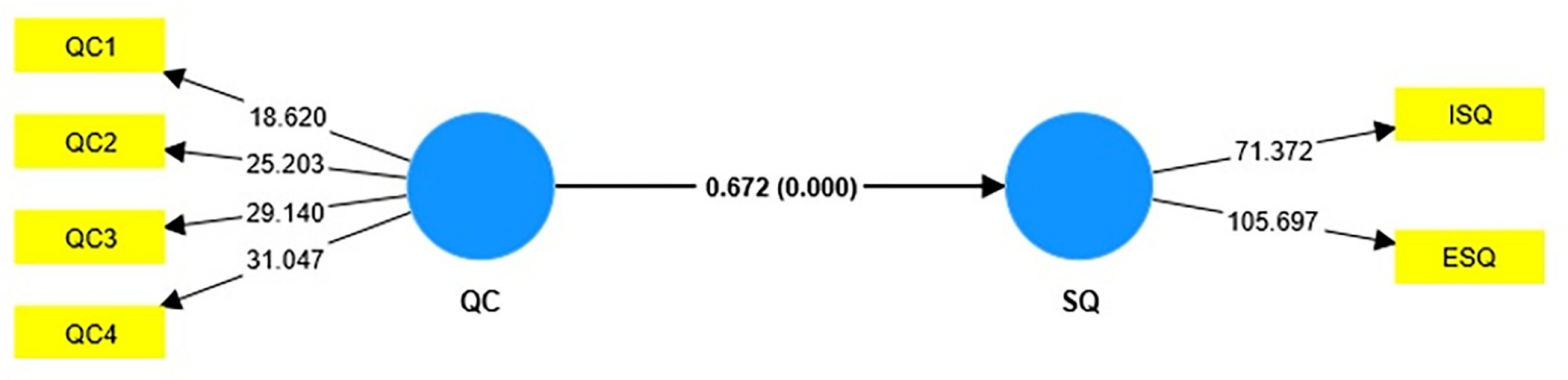
Structural model assessment.

The fifth hypothesis (H5) to address RQ2 was to examine the effect of overall QC on overall SQ in Pakistani (public and private) universities. To this end, PLS-SEM and SPSS were used to test the proposed hypothesis (H5) and determine the role of QC in predicting SQ through structural model evaluation (Fig 2). The PLS-SEM results showed that overall QC has a significant effect on overall SQ in the context of Pakistani universities (β = 0.672, t = 12.712, p = 0.000). Therefore, H5 was also accepted.

### 4.8 Regression analysis (Control variables)

In addition to the SEM analysis, a regression analysis was performed by adding control variables including sector, province, department, gender, age, educational level, and designation, so that the effect of QC on SQ can be accurately measured. “Control variables are the variables (i.e., factors, elements) that researchers seek to keep constant when conducting research” [113]. Control variables such as sector, province, and department were added considering suggestions from previous studies in which researchers included the type of property (public, private and mixed) as a control variable due to its potential effect on the environmental performance of organizations [114]. Similarly, some individual-level elements were also added as control variables, including gender, age, education level, and respondent designation, as indicated by previous researchers [115]. The results of the regression analysis (see Table 7) showed that all the control variables (sector, province, department, gender, age, education, and designation) explain 16.6% of the variance in SQ, while the “independent variable” QC explains 47.8% of the variance in the “dependent variable” SQ. In a nutshell, even after adding control variables, the values of β, t, and R^2^ decreased slightly, but the results still showed the significant effect of overall QC on the overall SQ in the context of Pakistani universities (β = 0.629, t = 7.576, p = 0.000). Therefore, H5 was also accepted.

**Table 7:** Results of regression analysis for service quality.

Predictor	Service Quality (SQ)		
	*B*	*R* ^*2*^	*ΔR* ^*2*^
Step 1			
*Control Variables		.166	
Step 2			
Quality Culture (QC)	.629	.478	0.312***

*Control Variables (sector, province, department, gender, age, education, designation)

***p < .001

## 5 Discussion and conclusion

### 5.1 Discussion on key findings

This study aimed to examine the applicability of RBV theory in the context of higher education by examining the possible effect of QC on SQ in Pakistani universities. To test the RVB theory, a simple yet effective research model was conceptualized based on gaps identified in previous literature. Following the principle of parsimony, the model introduced QC as a (valuable resource) and predictor variable, and SQ as a dependent variable. Thus, current research can guide future researchers in understanding the usefulness of QC as a valuable internal resource. The creation and promotion of QC can enhance the competitiveness of universities by providing quality education services to internal and external stakeholders.

Overall, five hypotheses were proposed, and all were found to be significant when tested. The results for hypotheses (H1 and H2) showed that there were significant differences in perceived QC and SQ between two types of universities (public and private) in Pakistan. However, the results demonstrated that the perceived QC and SQ levels of public universities are better than private universities in Pakistan. The results of this study differ from a recent study conducted in Kenya, which found that private universities had higher mean OC and SQ scores than public universities [89]. This may be for a couple of reasons: first, this study investigated OC in relation to SQ, which is a broader concept; however, in comparison, QC mainly emphasizes quality; second, the context of the study is also important as this study was conducted in a Kenyan context with a completely different culture.

Furthermore, two hypotheses (H3 and H4) were tested by simple linear regression, and the results demonstrated a significant effect of QC on SQ in both types of Pakistani universities (i.e., public, and private). However, the effect of QC on SQ was stronger (R^2^ = 0.611) in the case of private universities than in public universities where it was comparatively weaker (R^2^ = 0.212).

Finally, the hypothesis (H5) was tested by PLS-SEM, and the results revealed that the overall QC had a significant effect on the overall SQ of Pakistani universities. This result is also supported by some earlier studies in which researchers found a significant effect of OC on SQ in the public sector [9], the ICT sector [12], the power sector [10], and in the HE sector in Kenya [89]. However, the effect of QC on SQ is much stronger in the HE context of Pakistan than that of OC on SQ in the Kenyan HE context. This may be because this study attempted to examine the effect of QC rather than OC on SQ. QC is considered a subset of the broader concept of OC, whereas QC is a more specific and focused approach in terms of shared values, beliefs, and attitudes related to quality practices in an organization.

The cultural aspect cannot be ignored in any organizational context, and HE is no exception. In previous literature, researchers have stated various benefits of culture within the context of organizations, such as (1) it increases employee commitment and loyalty, (2) it enables the achievement of long-term goals, (3) it facilitates decision-making (4) saves time, (5) facilitates communication between employees, and (6) gives meaning and purpose to work [65]. The present study empirically confirms the considerable effect of QC (as a valuable university resource) on SQ in the Pakistani context. QC can enable universities to engage staff at different levels with quality as their primary goal and provide a variety of academic and non-academic services to different stakeholders. The increased emphasis on QC will raise quality awareness among staff in their daily activities, leading to improved performance of their universities.

### 5.2 Theoretical implications

This study provides several new insights and extends current knowledge in various ways. First, this study investigates the current state of perceived QC and SQ knowledge and practice and compares them in university settings (public and private), which is lacking in the existing literature. Although few studies attempted to investigate the effect of OC on SQ and found a significant relationship [8, 10, 116, 117], however, OC is a broader concept than QC, which is very specific. Therefore, the effect of QC on SQ has not been explored in an organizational setting, so this study filled a theoretical gap. Second, the study operationalizes SQ from a dual perspective, including ISQ (offered to university staff, including academic and non-academic staff), and ESQ (offered to students and the community). Despite the importance of the dual perspective of SQ, only a few researchers have examined both aspects of service [59–63]; however, so far there is no evidence that researchers have attempted to measure SQ from the perspective of internal and external customers in an educational context, so this study fills this gap. Finally, some researchers have studied QC as a predictor of organizational performance in manufacturing and service industries (including university settings); however, no studies have examined QC as a predictor of SQ in higher education settings. This study is unique in that it examines QC as a predictor of SQ in a university setting based on the perceptions of quality and administrative managers. These managers are considered part of the top management and are responsible for improving the productivity and performance of organizations by creating and promoting a culture of quality in their respective organizations.

### 5.3 Practical/Managerial implications

The study provides university leaders, quality and administrative managers, and policymakers with a solid statistical foundation to emphasize the fundamental role of QC by fostering quality awareness at all levels and departments of their respective universities. This will improve the quality of service provided to internal and external customers. Furthermore, administrative managers, in collaboration with the quality manager of the university, must develop policies and procedures to improve the quality of internal services provided to customers (employees) within the organization. This will standardize various processes and create consistency in the delivery of quality services, thus reducing customer dissatisfaction. This also applies to external customers (students, parents, employers, etc.) to enhance their experience of the services provided by the university or other organizations. This study also provides an opportunity for quality managers to develop an effective quality system for their organization with greater focus on internal and external stakeholder satisfaction and to increase quality awareness at all levels through regular audits. This will improve SQ and lead to higher levels of organizational performance.

### 5.4 Limitations and future research

Despite making valuable contributions, this study has the following limitations. First, the data were collected from administrative and quality managers, by ignoring teachers and students. Therefore, future researchers might include teachers and students in their studies. Second, following the principle of parsimony, the study employed a simple model with two variables (QC as an independent variable and SQ as a dependent variable). The aim was to introduce an effective predictor of SQ, and QC has been found to be a strong predictor of SQ in university settings. However, in the future, some other variables can be added to predict SQ in different sectors. Third, the study was cross-sectional in nature, with a relatively small sample size in a university setting. Therefore, future researchers may conduct longitudinal studies with larger populations and sample sizes in other sectors to improve the generalizability of the results.

### 5.5 Conclusion

The current study aimed to accomplish two objectives, including identifying differences in perceived QC and SQ between public and private universities based on the perceptions of administrative and quality managers; and determining the effect of perceived QC on SQ in both types of universities. The first objective was realized by proposing and testing two hypotheses. The first hypothesis was about identifying the difference in the perceived QC between public and private universities, and the results revealed substantial differences in the perceived QC in both types of universities. Likewise, the second hypothesis was about identifying differences in perceived SQ between public and private universities, and the results showed significant differences in perceived SQ for both types of universities. The results also suggest that the perceived QC and SQ scores (mean and median) are higher in public universities than in private universities in Pakistan. The second objective was achieved by testing three hypotheses, including the effect of QC on SQ in public and private universities separately and then collectively. The results of the three hypotheses showed that QC significantly affects individual and collective SQ in Pakistan’s public and private universities; however, this effect is stronger in private universities than in public universities. The study concludes that the higher the level of QC in an organization, the greater its impact on the organization’s SQ, leading to higher organizational performance.

## Supporting information

S1 File Survey data. (XLSX)

## References

[1] Gupta P, Kaushik N. Dimensions of service quality in higher education–critical review (students’ perspective). International Journal of Educational Management. 2018;32(4):580–605.

[2] Nygren-Landgärds C, Mårtensson LB, Pyykkö R, Bjørnestad JO, von Schoultz R. Quality culture at Nordic Universities. European Journal of Higher Education [Internet]. 2022;1–20. Available from: doi: 10.1080/21568235.2022.2116066

[3] Trewin D. The Importance of a Quality Culture. Statistics Canada. 2002;28(2):125–33.

[4] Ali HM, Musah MB. Investigation of Malaysian higher education quality culture and workforce performance. Quality Assurance in Education. 2012;20(3):289–309.

[5] Koskei R, Katwalo AM, Asienga I. Influence of Quality Culture on Performance of Research Institutions in Kenya. African journal of business and economic research [Internet]. 2015;10(1):25–54. Available from: https://hdl.handle.net/10520/EJC171071

[6] Wu SJ. The impact of quality culture on quality management practices and performance in Chinese manufacturing firms. International Journal of Quality & Reliability Management. 2015;32(8):799–814.

[7] Yusof AA, Ali J. Managing culture in organization. Malaysian Management Journal. 2000;35(2):60–5.

[8] Sarhan NM, Al Shishany A. The effect of culture on accommodation service quality perception and expectations. Management Science Letters. 2020;10(14):3357–66.

[9] Marzuki, Lawelai H, Sadat A, Nastia. The Impact of Organizational Culture on Public Service Quality in the Baubau City Regional Secretariat. Journal of Reserach Trends in Social Sciences and Humanities. 2022;1(1):52–9.

[10] Gantsho Y, Sukdeo N. Impact of organizational culture on service quality. In: Proceedings of the International Conference on Industrial Engineering and Operations Management [Internet]. Paris, France; 2018. p. 1659–67. Available from: http://www.ieomsociety.org/paris2018/papers/319.pdf

[11] Rabaa’i AA, Gammack JG. Effect of culture on perceptions of IS service quality. International Journal Intercultural Information Management. 2014;4(1):15–33.

[12] Metz D, Ilieş L, Nistor RL. The impact of organizational culture on customer service effectiveness from a sustainability perspective. Sustainability (Switzerland). 2020;12(15):1–27.

[13] Iqbal S, Ashfaq T, Taib CAB. A systematic literature review on organizational performance in global higher education: an affinity diagram approach. Pakistan Journal of Social Research. 2022;4(1):688–701.

[14] Banuri T. Does Pakistan’s Higher Education System Need Reform? Educationist Tariq Banuri [Internet]. TCM Originals. Pakistan; 2021 [cited 2021 Apr 8]. Available from: https://www.youtube.com/watch?v=lPkv9hEIUJw

[15] Hoodbhoy P. Pakistan’s higher education system—What went wrong and how to fix it. Pakistan Development Review. 2009;48(4):581–94.

[16] Nisar A. Challenges for Higher Education System in Pakistan. Pakistan & Gulf Economist [Internet]. 2019 Jan 21; Available from: https://www.pakistaneconomist.com/2019/01/21/challenges-for-higher-education-system-in-pakistan/

[17] Mastoi AG, Hai LX, Saengkrod W. Higher Education Service Quality Based on Students’ Satisfaction in Pakistan. European Scientific Journal ESJ. 2019;15(11).

[18] Iqbal S, Ashfaq T, Moosa K. Students’ perceived quality of academic programs in higher education institutions: an empirical study. Pakistan Journal of Educational Research [Internet]. 2022;5(4):1–22. Available from: https://pjer.org/index.php/pjer/article/view/645

[19] ISO 21001. ISO 21001:2018 (en) Educational Organizations—Management systems for educational organizations—Requirements with guidance for use [Internet]. International Organization for Standardization. 2018 [cited 2021 Dec 7]. Available from: https://www.iso.org/obp/ui/#iso:std:iso:21001:ed-1:v1:en

[20] Ali F, Zhou Y, Hussain K, Nair PK, Ragavan NA. Does higher education service quality effect student satisfaction, image and loyalty? A study of international students in Malaysian public universities. Quality Assurance in Education [Internet]. 2016;24(1):70–94. Available from: 10.1108/QAE-02-2014-0008

[21] Hasan M, Hosen MZ. The Quality of University Service: Its Impact on Students’ Satisfaction and Loyalty in Bangladesh State University. International Journal of Asian Education. 2020;1(3):135–46.

[22] Chandra T, Ng M, Chandra S, Priyono. The effect of service quality on student satisfaction and student loyalty: An empirical study. Journal of Social Studies Education Research. 2018;9(3):109–31.

[23] Bakrie M, Sujanto B, Rugaiyah R. The Influence of Service Quality, Institutional Reputation, Students’ Satisfaction on Students’ Loyalty in Higher Education Institution. International Journal for Educational and Vocational Studies. 2019;1(5):379–91.

[24] Banahene S, Kraa JJ, Kasu PA. Impact of HEdPERF on Students’ Satisfaction and Academic Performance in Ghanaian Universities; Mediating Role of Attitude towards Learning. Open Journal of Social Sciences. 2018;06:96–119.

[25] Annamdevula S, Bellamkonda RS. The effects of service quality on student loyalty: the mediating role of student satisfaction. Journal of Modelling in Management. 2016;11(2):446–62.

[26] Cheruiyot TK, Maru LC. Service quality and relative performance of public universities in East Africa. TQM Journal. 2013;25(5):533–46.

[27] Sumardi Fernandes AAR. The mediating effect of service quality and organizational commitment on the effect of management process alignment on higher education performance in Makassar, Indonesia. Journal of Organizational Change Management. 2018;31(2):410–25.

[28] Sinawi AS, Sharma S. Mediation Effects of Service Performance and Concerns of Customers on High Performance Work Systems and Institutional Performance in Higher Education Institutes. MIER Journal of Educational Studies, Trends & Practices. 2020;10(2):220–35.

[29] Parasuraman A, Zeithaml VA, Berry LL. A Conceptual Model of Service Quality and Its Implications for Future Research. Journal of Marketing. 1985;49(4):41–50.

[30] Smith AM. Some problems when adopting Churchill’s paradigm for the development of service quality measurement scales. Journal of Business Research. 1999;46(2):109–20.

[31] Caruana A, Ewing MT, Ramaseshan B. Assessment of the three-column format SERVQUAL: An experimental approach. Journal of Business Research. 2000;49(1):57–65.

[32] Athanassopoulos AD. Customer satisfaction cues to support market segmentation and explain switching behavior. Journal of Business Research. 2000;47(3):191–207.

[33] Mahapatra SS, Khan MS. A neural network approach for assessing quality in technical education: An empirical study. International Journal of Productivity and Quality Management. 2007;2(3):287–306.

[34] Harvey L, Green D. Defining Quality. Assessment & Evaluation in Higher Education [Internet]. 1993;18(1):9–34. Available from: 10.1080/0260293930180102

[35] Quinn A, Lemay G, Larsen P, Johnson DM. Service quality in higher education. Total Quality Management. 2009;20(2):139–52.

[36] Srikanthan G, Dalrymple J. Developing alternative perspectives for quality in higher education. International Journal of Educational Management. 2003;17(3):126–36.

[37] Shostack GL. Breaking free from product marketing. Journal of Allergy and Clinical Immunology. 1977;130(2):73–80.

[38] Shank MD, Walker M, Hayes T. Understanding professional service expectations: Do we know what our students expect in a quality education? Journal of Professional Services Marketing. 1995;13(1):71–89.

[39] Harrington HJ. Business Process Improvement: The Breakthrough Strategy for Total Quality, Productivity, and Competitiveness. New York: McGraw-Hill; 1991.

[40] Sallis E. Total Quality Management in Education. 3rd ed. Londan, UK: Kogan Page Ltd.; 2002. 1–163 p.

[41] Xie D. Exploring organizational learning culture, job satisfaction, motivation to learn, organizational commitment, and internal service quality in a sport organization [Internet]. The Ohio State University; 2005. Available from: http://journal.um-surabaya.ac.id/index.php/JKM/article/view/2203

[42] Zeithaml V, Bitner MJ, Gremler DD. Services Marketing: Integrating Customer Focus Across the Firm. Seventh. Vol. 51. McGraw-Hill Education; 2018. 163–168 p.

[43] Dauda A, Maishanu MM, Mawoli MA. Effect of Internal Service Quality on Employee Job Satisfaction: Evidence from Abubakar Gimba Library, IBB University, Lapai–Nigeria. American International Journal of Contemporary Research. 2013;3(6):88–96.

[44] Latif KF, Baloch QB, Rehman SU. Role of Internal Service Quality (ISQ) in the relationship between Internal Marketing and Organizational Performance. City University Research Journal. 2016;6(1):01–22.

[45] Skarpeta K, Koemtzi M, Aidonis D. Measuring internal service quality: the case of the Greek public higher education institutions. TQM Journal. 2020;32(2):268–87.

[46] Czepiel JA. Service encounters and service relationships: Implications for research. Journal of Business Research. 1990;20(1):13–21.

[47] Parasuraman A, Zeithaml VA, Berry LL. SERVQUAL: A Multiple-Item Scale For Measuring Consumer Perceptions of Service Quality. Journal of Retailing. 1988;64(1):12–40.

[48] Carman JM. Consumer perceptions of service quality: an assessment of the SERVQUAL dimensions. Journal of Retailing [Internet]. 1990;66:33–55. Available from: doi: 10.1016/j.jaci.2012.05.050

[49] Cronin JJ, Taylor SA. Measuring Service Quality: A Reexamination and Extension. Journal of Marketing. 1992;56(3):55–68.

[50] Babakus E, Boller GW. An Empirical Assessment of the SERVQUAL Scale. Journal of Business Research. 1992;24:253–68.

[51] Saliba K, Zoran AG. Measuring Higher Education Services Using the SERVQUAL Model. Journal of Universal Excellence,. 2018;4:160–79.

[52] Sohail MS, Hasan M. Students’ perceptions of service quality in Saudi universities: the SERVPERF model. Learning and Teaching in Higher Education: Gulf Perspectives. 2021;17(1):54–66.

[53] Campos DF, Santos GS d., Castro FN. Measuring students’ expectations of service quality of a higher education institution in a longitudinal design. International Journal of Services and Operations Management. 2018;31(3):303–24.

[54] Yılmaz K, Temizkan V. The Effects of Educational Service Quality and Socio-Cultural Adaptation Difficulties on International Students’ Higher Education Satisfaction. SAGE Open. 2022;12(1):1–18.

[55] Mwiya B, Bwalya J, Siachinji B, Sikombe S, Chanda H, Chawala M. Higher Education Quality and Student Satisfaction Nexus: Evidence from Zambia. Creative Education. 2017;08(07):1044–68.

[56] Dužević I, Časni AC. Student and faculty perceptions of service quality: the moderating role of the institutional aspects. Higher Education. 2015;70(3):567–84.

[57] Wright C, O’Neill M. Service quality evaluation in the higher education sector: An empirical investigation of students’ perceptions. Higher Education Research and Development. 2002;21(1):23–39.

[58] Mwiya B, Siachinji B, Bwalya J, Sikombe S, Chawala M, Chanda H, et al. Are there study mode differences in perceptions of university education service quality? Evidence from Zambia. Cogent Business and Management [Internet]. 2019;6(1):1–19. Available from: 10.1080/23311975.2019.1579414

[59] Tam JLM, Wong YH. Interactive selling: A dynamic framework for services. Journal of Services Marketing. 2001;15(5):379–96.

[60] Chow-Chua C, Komaran R. Managing service quality by combining voice of the service provider and voice of their customers. Managing Service Quality: An International Journal. 2002;12(2):77–86.

[61] Dedeke A. Service quality: A fulfilment-oriented and interactions-centred approach. Managing Service Quality: An International Journal. 2003;13(4):276–89.

[62] Svensson G. A customized construct of sequential service quality in service encounter chains: time, context, and performance threshold. Managing Service Quality: An International Journal. 2004;14(6):468–75.

[63] Svensson G. New aspects of research into service encounters and service quality. International Journal of Service Industry Management. 2006;17(3):245–57.

[64] Dellana SA, Hauser RD. Toward defining the quality culture. Engineering Management Journal. 1999;11(2):11–5.

[65] Malhi RS. Creating and Sustaining: A Quality Culture. Journal of Defense Management. 2013;s3(002):1–4.

[66] EUA. Quality Culture in European Universities: A Bottom-Up Approach [Internet]. Brussels, Belgium; 2006. Available from: http://www.eua.be/eua/jsp/en/upload/Quality_Culture_2002_2003.1150459570109.pdf

[67] Vlăsceanu L, Grünberg L, Pârlea D. Quality Assurance and Accreditation: A Glossary of Basic Terms and Definitions [Internet]. Second. Seto M, Wells PJ, editors. Bucharest: UNESCO-CEPES 2007; 2007. 117 p. Available from: https://unesdoc.unesco.org/ark:/48223/pf0000134621

[68] Adina-Petruţa P. Quality Culture—A Key Issue for Romanian Higher Education. Procedia—Social and Behavioral Sciences. 2014;116:3805–10.

[69] Edvardsson B, Olsson J. Key concepts for new service development. The Service Industries Journal. 1996;16(2):140–64.

[70] Teas RK. Expectations, Performance Evaluation, and Consumers’ Perceptions of Quality. Journal of Marketing. 1993;57(4):18–34.

[71] Marshall GW, Baker J, Finn DW. Exploring internal customer service quality. Journal of Business and Industrial Marketing. 1998;13(4/5):381–92.

[72] Chiang CF, Wu KP. The influences of internal service quality and job standardization on job satisfaction with supports as mediators: flight attendants at branch workplace. International Journal of Human Resource Management. 2014;25(19):2644–66.

[73] Nagel PJA, Cilliers WW. Customer Satisfaction: A Comprehensive Approach. International Journal of Physical Distribution & Logistics Management. 1990;20(6):2–46.

[74] Chen C-F, Kao Y-L. Investigating the antecedents and consequences of burnout and isolation among flight attendants. Tourism Management [Internet]. 2012;33(4):868–74. Available from: 10.1016/j.tourman.2011.09.008

[75] Jun M, Cai S. Examining the relationships between internal service quality and its dimensions, and internal customer satisfaction. Total Quality Management and Business Excellence. 2010;21(2):205–23.

[76] Xuan Y, Yunchen W. The Impact of Commercial Banks’ Internal Service Quality on the Front-Line Employees’ Service Performance: Based on the Perspective of Internal Marketing. In: 2015 International Conference on Service Science, ICSS [Internet]. 2015. p. 70–4. Available from: https://ieeexplore.ieee.org/document/7400774

[77] Moslehpour M, Chau KY, Zheng JJ, Hanjani AN, Hoang M. The mediating role of international student satisfaction in the influence of higher education service quality on institutional reputation in Taiwan. International Journal of Engineering Business Management [Internet]. 2020;12(100):1–16. Available from: 10.1177/1847979020971955

[78] Handayanto E. Mediating Role of Satisfaction on Relationship between Service Quality and Word of Mouth in Islamic Private Universities in Indonesia. 2018;231(Amca):530–4.

[79] Hwarng HB, Teo C. Translating customers’ voices into operations requirements: A QFD application in higher education. International Journal of Quality and Reliability Management. 2001;18(2):195–225.

[80] de Jager J, Gbadamosi G. Predicting students ‘ satisfaction through service quality in higher education. The International Journal of Management Education [Internet]. 2013;11(3):107–18. Available from: 10.1016/j.ijme.2013.09.001

[81] Owlia MS, Aspinwall EM. A framework for the dimensions of quality in higher education. Quality Assurance in Education. 1996;4(2):12–20.

[82] Nicolescu L. Applying marketing to higher education: Scope and limits. Management & Marketing. 2009;4(2):35–44.

[83] Joseph M, Joseph B. Employers’ Perceptions of Service Quality in Higher Education. Journal of Marketing for Higher Education. 1997;8(2):1–13.

[84] Senthilkumar N, Arulraj A. SQM-HEI–determination of service quality measurement of higher education in India. Journal of Modelling in Management. 2011;6(1):60–78.

[85] Teeroovengadum V, Kamalanabhan TJ, Seebaluck AK. Measuring service quality in higher education: Development of a hierarchical model (HESQUAL). Quality Assurance in Education. 2016;24(2):244–58.

[86] Hama J, Bawais T, Sagsan M, Ertugan A. The Impact of Service Quality on Student and Academic Staff Satisfaction within Higher Education Institutions: A Case Study of Sulaimani City in Northern Iraq. Revista Argentina De Clínica Psicológica. 2020;XXIX(5):440–52.

[87] Khalid S., Ali KAM, Makhbul ZKM. Assessing The Effect of Higher Education Service Quality on Job Satisfaction Among Lecturers in Premier Polytechnics HEdPERF Model. Scientific Journal of Logistics [Internet]. 2019;15(3):425–36. Available from: 10.17270/J.LOG.2019.356

[88] Chang H-T, Chou Y-J, Miao M-C, Liou J-W. The effects of leadership style on service quality: enrichment or depletion of innovation behaviour and job standardisation. Total Quality Management and Business Excellence [Internet]. 2019;1–17. Available from: 10.1080/14783363.2019.1626708

[89] Ngugi DW, Gachunga H, Mukanzi C. A comparative analysis on the relationship between organizational culture and service quality in public and private universities in Kenya. Human Resource and Leadership Journal. 2021;6(1):1–15.

[90] Barney JB. Firm Resources and Sustained Competitive Advantage. Journal of Management. 1991;17(1):99–120.

[91] Wernerfelt B. A Resource-based View of the Firm. Strategic Management Journal. 1984;5(2):171–80.

[92] Hair JF, Risher JJ, Sarstedt M, Ringle CM. When to use and how to report the results of PLS-SEM. European Business Review. 2019;31(1):2–24.

[93] Dolma S. The central role of the unit of analysis concept in research design. Istanbul University Journal of the School of Business Administration. 2010;39(1):169–74.

[94] Kumar S. Understanding Different Issues of Unit of Analysis in a Business Research. Journal of General Management Research. 2018;5(2):70–82.

[95] Rezaei G, Mardani A, Senin AA, Wong KY, Sadeghi L, Najmi M, et al. Relationship between culture of excellence and organisational performance in Iranian manufacturing companies. Total Quality Management and Business Excellence. 2016;29(1–2):94–115.

[96] Huong PT. Quality culture of a faculty in a Vietnamese university. Ho Chi Minh City Open University Journal of Science. 2018;8(2):34–53.

[97] Abubakar A, Ahmed S. The Effect of a Transformational Leadership Style on the Performance of Universities in Nigeria. Pakistan Journal of Educational Research and Evaluation. 2017;2(1):59–76.

[98] Hilman H, Abubakar A, Kaliapan N. The effect of quality culture on university performance. Journal of Business and Retail Management Research. 2017;11(4):25–33.

[99] Krejcie R V., Morgan DW. Determining Sample Size for Research Activities. Educational and Psychological Measurement. 1970;30:607–10.

[100] Arnab R. Survey Sampling: Theory and Applications. Academic Press; 2017. 1–899 p.

[101] Asif M, Awan MU, Khan MK, Ahmad N. A model for total quality management in higher education. Quality and Quantity. 2013;47(4):1883–904.

[102] Asif M, Searcy C. A composite index for measuring performance in higher education institutions. International Journal of Quality and Reliability Management. 2014;31(9):983–1001.

[103] Hui CH, Cheng K, Gan Y. Psychological collectivism as a moderator of the impact of supervisor-subordinate personality similarity on employees’ service quality. Applied Psychology. 2003;52(2):175–92.

[104] Nedwek BP, Neal JE. Performance indicators and rational management tools: A comparative assessment of projects in North America and Europe. Research in Higher Education. 1994;35(1):75–103.

[105] Badri MA, Abdulla MH. Awards of excellence in institutions of higher education: An AHP approach. International Journal of Educational Management. 2004;18(4):224–42.

[106] Podsakoff PM, MacKenzie SB, Podsakoff NP. Sources of method bias in social science research and recommendations on how to control it. Annual Review of Psychology. 2012;63:539–69. doi: 10.1146/annurev-psych-120710-100452 21838546

[107] Kock N. Common method bias in PLS-SEM: A Full Collinearity Assessment Approach. International Journal of e-Collaboration. 2015;11(4):1–10.

[108] Hair JF, Hult GTM, Ringle CM, Sarstedt M. A Primer on Partial Least Squares Structural Equation Modeling (PLS-SEM). Second. SAGE Publications, Inc; 2017. 374 p.

[109] Hair JF, Sarstedt M, Hopkins L, Kuppelwieser VG. Partial least squares structural equation modeling (PLS-SEM): An emerging tool in business research. European Business Review. 2014;26(2):106–21.

[110] McNeish D. Thanks Coefficient Alpha, We’ll Take It From Here. Psychological Methods [Internet]. 2018;23(3):412–33. Available from: doi: 10.1037/met0000144 28557467

[111] Wasko MM, Faraj S. Why should I share? Examining social capital and knowledge contribution in electronic networks of practice. MIS Quarterly. 2005;29(1):35–57.

[112] Henseler J, Ringle CM, Sarstedt M. A new criterion for assessing discriminant validity in variance-based structural equation modeling. Journal of the Academy of Marketing Science. 2015;43(1):115–35.

[113] Allen M. The SAGE Encyclopedia of Communication Research Methods. Vol. 1. SAGE Publications, Inc.; 2017. 1969 p.

[114] Paillé P, Chen Y, Boiral O, Jin J. The Impact of Human Resource Management on Environmental Performance: An Employee-Level Study. Journal of Business Ethics. 2014;121(3):451–66.

[115] Obeidat SM, Al Bakri AA, Elbanna S. Leveraging “Green” Human Resource Practices to Enable Environmental and Organizational Performance: Evidence from the Qatari Oil and Gas Industry. Journal of Business Ethics [Internet]. 2020;164(2):371–88. Available from: 10.1007/s10551-018-4075-z

[116] Tsoukatos E. Impact of Culture on Service Quality: What We Know and What We Need to Learn. International Consumer Behavior: A Mosaic of Eclective Perspectives- Handbook on International Consumer Behavior. 2011;20–36.

[117] Furrer O, Liu BSC, Sudharshan D. The Relationships between Culture and Service Quality Perceptions: Basis for Cross-Cultural Market Segmentation and Resource Allocation. Journal of Service Research. 2000;2(4):355–71.

